# Henry Bliss Noble, D.D.S.

**Published:** 1902-04

**Authors:** 


### Obituary.

### HENRY BLISS NOBLE, D.D.S.

At a special meeting called for the purpose, the Board of Dental Examiners for the District of Columbia, by a unanimous rising vote, adopted the following resolutions:

Whereas, Death has suddenly bereaved us of our most beloved and distinguished associate, Dr. Henry Bliss Noble.

Whereas, In his relations with us he was the soul of honor, while always kind, genial, generous, and helpful,—a man of unselfish disposition who ever labored to advance the best interests of all.

*Resolved,* That this Board, cherishing his memory gratefully, and keenly feeling his loss, desire to spread this expression of their appreciation upon their records.

*Resolved,* That a copy of these resolutions be transmitted to his family and the dental journals.

M. F. Finley.
C. W. Appler.
H. J. Allen.
J. H. London.

